# Liming-Induced Nitrous Oxide Emissions from Acidic Soils Dominated by Stimulative Nitrification

**DOI:** 10.3390/biology14091110

**Published:** 2025-08-22

**Authors:** Xiaoxiao Xiang, Hongyang Gong, Waqar Ahmed, Rodney B. Thompson, Wenxuan Shi, Junhui Yin, Qing Chen

**Affiliations:** 1 College of Resources and Environmental Sciences, China Agricultural University, Beijing 100193, China; 2 Department of Agronomy, University of Almeria, Carretera de Sacramento s/n, La Cañada de San Urbano, 04120 Almería, Spain; 3 Environmental Research Centre, Teagasc, Johnstown Castle, Y35 TC97 Wexford, Ireland; 4 State Key Lab of Biocontrol, Guangdong Provincial Key Laboratory of Plant Stress Biology, School of Agriculture and Biotechnology, Shenzhen Campus of Sun Yat-sen University, Shenzhen 518107, China

**Keywords:** soil amendments, acidic soil, nitrous oxide, ammonia-oxidizing bacteria

## Abstract

Nitrous oxide (N_2_O) is a potent greenhouse gas, with emissions occurring mostly from agricultural soils, especially acidic soils. This research aimed to elucidate the response of soils dominated by nitrification-driven N_2_O production to alkaline amendments, given that nitrification is a key process in N_2_O emission. This study investigated the impact of an alkaline mineral amendment (CSMP) on N_2_O emission, nitrification rate, and functional gene abundance. Using a robotic automated incubation system, CSMP both alone and in combination with urea was applied to two acidic soils (CL: pH 5.81; WS: pH 4.91). The results demonstrated that, relative to the CK, the CSMP-only treatment significantly increased N_2_O emissions by 18.4-fold in these acidic soils, with a 61.6-fold increase in the U + CSMP treatment. This very large increase was driven by a rise in AOB-*amoA* abundance and a concurrent decline in AOA-*amoA*, which was confirmed by structural equation modeling, which showed that the increase in pH strongly influenced N_2_O emission primarily through AOB-*amoA*. Although CSMP is effective for reversing soil acidification, its use must be carefully managed to prevent stimulation of N_2_O emissions. Future strategies should explore combining CSMP with approaches that can mitigate nitrification while maintaining its soil improvement benefits. This study provides critical insights for developing balanced management practices that address both soil health and climate change mitigation in acidic agricultural systems.

## Simple Summary

This study examined how liming influences nitrous oxide emissions by increasing pH in acidic soils. The results showed that soil nitrous oxide emissions increased following alkaline amendment, especially when combined with urea addition, by stimulating nitrification. These findings indicated that while liming alleviates soil acidity, it can also increase greenhouse gas emissions. Future work is suggested to develop optimal management of alkaline amendments for soil pH management while restricting nitrous oxide emissions.

## 1. Introduction

N_2_O is a potent greenhouse gas that is also projected to remain as the dominant anthropogenic ozone-depleting agent this century [1,2,3]. Modern crop production systems, relying on intensive N fertilization to sustain high yields, represent the largest anthropogenic source of global N_2_O emissions [4,5,6,7], with atmospheric concentrations increasing at an annual rate of 0.75–1.0 ppb [8]. N_2_O emissions are generally appreciably larger from acidic compared with alkaline soils [9]. Emission factors (EFs) of N_2_O from acidic soils are more than three times those from alkaline soils [10,11]. Consequently, mitigating N_2_O emissions from acidic agricultural soils is an urgent priority.

Alkaline amendments have long been used to deal with low-pH soils. While effectively addressing soil acidification, these amendments can concurrently affect N_2_O emissions [12,13,14]. These emissions are primarily regulated by soil pH [15,16,17]. A meta-analysis of 121 field studies reported an average reduction of 21.3% in N_2_O emission following liming [18]. This reduction was primarily attributed to pH-enhanced abundance of the bacterial N_2_O reductase enzyme (*nosZ*), the sole known biological sink for N_2_O, which is impaired under low pH [19,20,21] and which reduces the N_2_O/N_2_ ratio during denitrification [22,23,24]. Additionally, the suppression of acid-tolerant fungal denitrification further enhances the mitigation effect at higher pH [25,26].

However, there are reports that indicate that alkalizing agents can have the opposite effect and can stimulate N_2_O emission [23,27]. This stimulatory effect can result from enhanced nitrification [23,28,29] and elevated nitrate (NO_3_^−^) availability fueling nitrification-derived N_2_O formation [30,31] under alkaline conditions. This apparent contradiction occurs because of the opposing influence of pH on distinct N_2_O production pathways: while elevated pH enhances the capacity for bacterial N_2_O consumption [24,32], it can also promote the abundance of nitrifiers, particularly ammonia-oxidizing bacteria (AOB) [33,34]. Although nitrification was traditionally regarded as restricted in acidic soils due to limited ammonium (NH_4_^+^) availability [35], recent findings demonstrate significant nitrification-derived N_2_O fluxes under acidic conditions [23,27,36]. A comprehensive global analysis of 5438 observations identified a distinct hump-shaped relationship between soil pH and N_2_O EFs, peaking in acidic soils [10]. This demonstrates nitrification as a major source of N_2_O across a wider pH range than previously assumed, highlighting the complex interplay between pH and both nitrification and denitrification processes governing net N_2_O flux.

Consequently, the net impact of pH amendment on N_2_O emissions is contingent upon which N_2_O production pathway is dominant. This study hypothesized that (i) in acidic soils dominated by nitrification, application of the CSMP alkaline amendment will significantly increase N_2_O emission; (ii) co-application of CSMP with urea will further stimulate the increased N_2_O emission; and (iii) the stimulation of N_2_O emission is facilitated by an increased population of ammonium oxidizers (AOA/AOB). To test these hypotheses, this study employed an alkaline amendment (CSMP) in two different acidic soils [37].

## 2. Materials and Methods

### 2.1. Soil Sampling and Physicochemical Characteristics

In the context of this research, topsoil samples (0–20 cm) were collected from two different sites (CL and WS) in Zhuzhou City, Hunan Province, China, with coordinates of 27°48′46″ N, 113°28′8″ E (CL) and 27°48′49″ N, 113°25′56″ E (WS). The WS soil (26.6 g kg^−1^) appears to be sandier and richer in organic carbon compared with the CL soil (18.6 g kg^−1^), which has a higher proportion of clay. The region falls within a subtropical humid monsoon climate zone, characterized by annual rainfall between 1200 and 1600 mm and a yearly average temperature of approximately 18 °C. Soil samples collected from the area were acidic, with pH values of 5.81 for CL and 4.91 for WS. Detailed information about the properties of the soils can be found in Table S1. Prior to incubation, the soil samples were passed through a 2 mm sieve and air-dried. Throughout the study, all measurements and data calculations were based on the oven-dried soil weight.

### 2.2. The Alkaline Mineral Amendment

The alkaline mineral additive employed in this study was obtained from Kingenta Ecological Engineering Group Co., Ltd., based in Linyi, Shandong Province, China. This product is composed of a blend of partially decomposed phosphate rock and insoluble potassium-containing silicate minerals, marketed commercially available as a Calcium–Silicon–Magnesium–Potassium (CSMP) product. It has been approved for agricultural applications and meets the national standards set forth in GB/T 36207–2018 [38]. The pH of the amendment was 10.3. The detailed composition is provided in Table S2.

### 2.3. Robotized Batch Cultivations for Respiratory Phenotype Experiment

Thirty grams of oven-dried equivalent soil was allocated to four different treatments, each performed in triplicate. The treatments were as follows: (i) without urea and CSMP (CK), (ii) urea added at 100 mg N per kilogram of soil (U), (iii) CSMP applied at 10 g per kilogram of soil (CSMP), and (iv) a combined treatment of 10 g kg^−1^ CSMP with 100 mg N kg^−1^ urea (U + CSMP). The urea dose (60 mg N kg^−1^ soil) was equivalent to a field application rate of 92 kg N ha^−1^. Similarly, the 10 g kg^−1^ soil CSMP dose was equivalent to a field application of 20 t ha^−1^ [39,40].

The urea, containing 46% nitrogen and obtained from Sinopharm Chemical Reagent Co., Ltd., Shanghai, China, was dissolved in deionized water before being applied [39,40]. The CSMP amendment was thoroughly mixed into the soil to ensure even distribution. Following addition of the amendments, the treated soil samples were transferred into 120 mL serum bottles, incubated at 60% water-holding capacity (WHC), and maintained at a steady 20 ± 0.5 °C for 30 d. To create airtight conditions, each bottle was gastight-sealed with bromobutyl rubber septa and aluminum caps sourced from Macherey-Nagel, GmbH & Co. KG, Düren, Germany. Bottle headspaces were purged with an oxygen–helium mixture (21% oxygen by volume) through five vacuum and refill cycles, which effectively eliminated any residual gases. Afterward, a syringe without a plunger filled with 5 mL of distilled water was inserted to balance the gas pressure to one atm [19].

Automated headspace gas sampling was performed at 0, 1, 3, 7, 15, and 30 days using a robotic incubation system described in a previous work [37]. Isostatic pressure maintenance was achieved by replenishing each sampled volume with ultra-pure helium (99.999%). The gases were drawn out with a peristaltic pump (Gilson Model 222, Gilson S.A.S., Villiers-le-Bel, France) and subsequently analyzed with an Agilent 7890A gas chromatograph (Agilent Technologies Inc., Santa Clara, CA, USA), which had both electron capture and thermal conductivity detectors. The entire sampling and analytical process was automatically controlled using a tailored Python 2.6.3 script. The gas emission was calculated using the method of Lars R. Bakken [41].

Six batches of parallel incubation were concurrently conducted for 0, 1, 3, 7, 15, and 30 days for a suite of analyses. The analyses included the determination of soil pH, ammonium nitrogen (NH_4_^+^-N), and nitrate nitrogen (NO_3_^−^-N); determination of the net nitrification rate; and identification of changes in functional gene populations.

### 2.4. Measurements and Analysis

#### 2.4.1. Chemical Analysis

Immediately following incubation, soil was extracted with a 1 mol L^−1^ KCl (Sinopharm Chemical Reagent Co., Ltd., Shanghai, China) solution at a 1:5 (*w*/*v*) soil-to-solution ratio. The concentrations of ammonium N (NH_4_^+^-N) and nitrate N (NO_3_^−^-N) in the extracts were determined using a continuous flow analyzer (model AA3, Seal Analytical, Norderstedt, Germany). The net nitrification rate (mg N kg^−1^ h^−1^) was calculated as follows:

(1) $$\mathrm{Net}\;\mathrm{nitrification}\;\mathrm{rate}\;=\;\frac{{\mathrm{NO}}_{3}^{-}\mathrm{-N}\;(\mathrm{AI})-{\mathrm{NO}}_{3}^{-}\mathrm{-N}\;(\mathrm{BI})}{\mathrm{incubation}\;\mathrm{time}},$$

where (AI) = after incubation, (BI) = before incubation.

#### 2.4.2. DNA Extraction and qPCR of Targeted Genes

Total DNA from the soil was isolated using the FastDNA^®^ Spin Kit (MP Biomedicals, Irvine, CA, USA) for Soil. DNA quality was verified through gel electrophoresis on a 1% agarose gel, while concentration of DNA and purity were measured with a NanoDrop 2000 spectrophotometer (Thermo Fisher Scientific Inc., Wilmington, NC, USA). To determine the abundance of specific functional genes, quantitative real-time PCR (qPCR) was employed.

The quantitative PCR (qPCR) reaction setup, totaling 20 μL, included the following ingredients: 4 μL of 5 × TransStart FastPfu (TransGen Biotech Co., Ltd., Beijing, China) Buffer, 2 μL of 2.5 mM dNTP mix, 0.8 μL each of forward and reverse primers at 5 μm concentration, 0.4 μL of TransStart FastPfu DNA polymerase, 10 ng of extracted template DNA, and enough sterile distilled water to bring the total volume to 20 μL. Each sample was analyzed in triplicate to ensure accuracy. The sequences of the primers employed are provided in Table S3. The thermal cycling protocol comprised an initial denaturation at 95 °C for 5 min, then 45 cycles of denaturation at 95 °C for 15 s, annealing at 55 °C for 30 s, and extension at 72 °C for 30 s.

### 2.5. Statistical Analysis

For statistical evaluation, differences across treatment groups were analyzed using one-way ANOVA with SPSS version 20.0 (IBM, Corporation, Armonk, NY, USA). Significant differences (*p* < 0.05) were identified by least significant difference (LSD) test. All visualizations were generated using Origin 2024 (OriginLab Corporation, Northampton, MA, USA). The lavaan package by R 4.3.1 (R Foundation for Statistical Computing, Vienna, Austria) was utilized for structural equation modeling (SEM) to assess the relationships between N_2_O emissions, soil properties, and gene abundances. The modification index (MI) was used to assess whether the inclusion of omitted pathways could optimize the initial conceptual model. The chi-square test (χ^2^, *p* > 0.05), comparative fit index (CFI > 0.90), and standardized root-mean-square residual (SRMR < 0.08) were employed to evaluate model fit.

## 3. Results

### 3.1. Soil N_2_O Emissions in Response to CSMP and Urea Application

**Figure 1:**
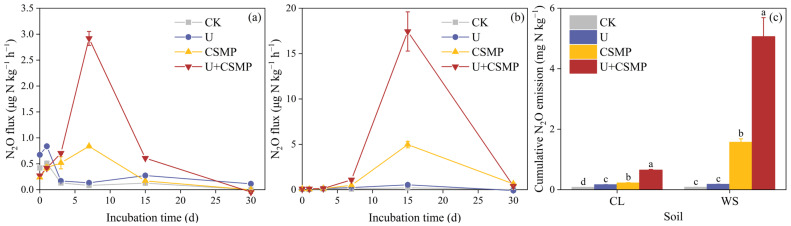
(**a**) N_2_O flux in soil CL, (**b**) N_2_O flux in soil WS, and (**c**) cumulative N_2_O production in both acidic soils CL and WS, all as influenced by CSMP and urea application. CK, control; U, urea; CSMP, Calcium–Silicon–Magnesium–Potassium fertilizer; U + CSMP, combined application of Calcium–Silicon–Magnesium–Potassium fertilizer and urea. Values are means ± SE (n = 3). Different lowercase letters indicate significant differences among treatments (*p* < 0.05).

The experimental results showed consistent temporal patterns in N_2_O emissions from the two acidic soils of an initial surge followed by a decline (Figure 1a,b). In soil CL, N_2_O peak values occurred on day 1 for CK (0.51 μg N kg^−1^ h^−1^) and U (0.84 μg N kg^−1^ h^−1^), while CSMP and U + CSMP peaked on day 7 (0.84 and 2.92 μg N kg^−1^ h^−1^, respectively). Conversely, soil WS displayed a peak in N_2_O emissions on day 15 across all treatments, with the U + CSMP treatment achieving the highest emission rate of 17.4 μg N kg^−1^ h^−1^.

Compared with the CK, the U treatment increased cumulative N_2_O emissions by 1.07-fold in CL soil and 1.21-fold in WS soil (*p* < 0.05; Figure 1c). The CSMP-only treatment raised emissions by 1.78-fold (CL) and 18.4-fold (WS) relative to CK (*p* < 0.05). The combined U + CSMP treatment further enhanced emissions, reaching 7.13-fold (CL) and 61.6-fold (WS) compared with CK, equivalent to 2.92-fold (CL) and 27.3-fold (WS) increases over the U treatment (*p* < 0.05).

### 3.2. Dynamics of NH_4_^+^-N and NO_3_^−^-N Concentrations and Net Nitrification Rate in Response to CSMP and Urea Application

**Figure 2:**
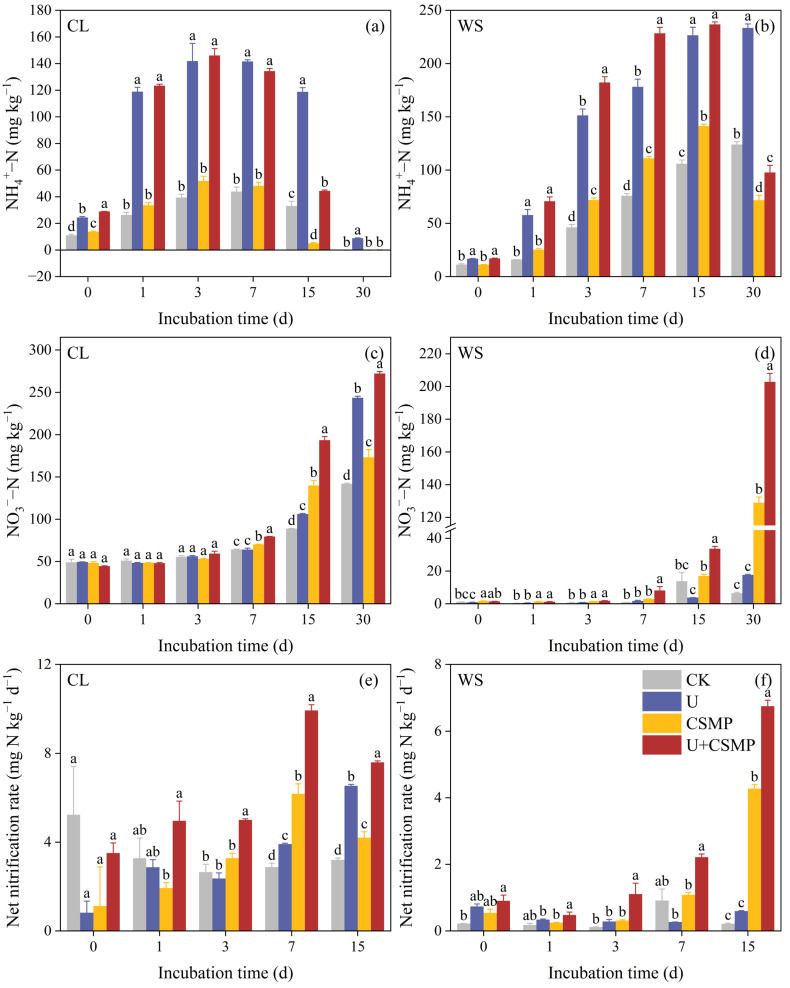
(**a**,**b**) Dynamics of NH_4_^+^-N concentrations; (**c**,**d**) NO_3_^−^-N concentrations; and (**e**,**f**) net nitrification rate in response to CSMP and urea application in two acidic soils (CL and WS). (**a**,**c**,**e**) represent soil CL, while (**b**,**d**,**f**) represent soil WS. CK, control; U, urea; CSMP, CSMP fertilizer; U + CSMP, combined application of CSMP fertilizer and urea. Data are presented as means ± SE (n = 3). Different lowercase letters indicate significant differences among treatments (*p* < 0.05).

During the incubation, NH_4_^+^-N concentrations in CL and WS soils followed a general pattern of initial increase and subsequent decline, which was substantially modulated by urea application (Figure 2a,b). In soil CL, NH_4_^+^-N peaked across all treatments on day 3, and then declined steadily. The CSMP treatment did not significantly alter NH_4_^+^-N concentrations in the initial 7 days; however, it significantly accelerated its reduction on day 15 relative to CK (*p* < 0.05). In soil WS, NH_4_^+^-N showed the same initial rise followed by decline. Relative to the CK treatment, the U treatment significantly increased NH_4_^+^-N (*p* < 0.05). The CSMP treatment further increased NH_4_^+^-N during the first 15 days (*p* < 0.05) but decreased it by day 30 (*p* < 0.05).

Throughout the incubation, NO_3_^−^-N concentrations in both CL and WS soils progressively increased in all treatments (Figure 2c,d). The U treatment, compared with CK, significantly enhanced cumulative NO_3_^−^-N concentration after 30 days in both soils, attaining 243 mg N kg^−1^ in CL and 18.6 mg N kg^−1^ in WS (*p* < 0.05). CSMP application substantially increased the 30-day cumulative NO_3_^−^-N concentrations in both soils compared with the CK treatment. Notably, the U + CSMP treatment, compared with CK, increased NO_3_^−^-N concentrations in WS soil by 9.94 times (*p* < 0.05).

The net nitrification rate in soil CL remained consistent throughout the incubation under the CK treatment, fluctuating between 2.65 and 5.23 mg N kg^−1^ d^−1^ (Figure 2e). Compared with CK, both U and CSMP treatments significantly increased the net nitrification rate, with the U + CSMP treatment showing the most substantial effect. In soil WS, the impact of all treatments on the net nitrification rate was most evident on day 30 (Figure 2f), where the CSMP treatment enhanced the rate by 19.1 times relative to CK, and the U + CSMP treatment resulted in a 10.3-fold increase relative to the U treatments.

### 3.3. Abundance of Functional Genes Related to N_2_O Production and Reduction

**Figure 3:**
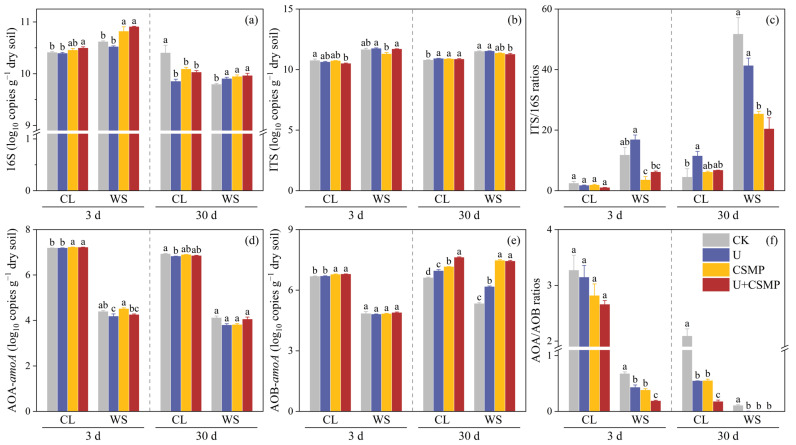
Effects of CSMP and urea application on microbial community structure in two acidic soils (CL and WS) over 3 d and 30 d periods. (**a**) Bacterial abundance, (**b**) fungal abundance, (**c**) fungal-to-bacterial ratio, (**d**) ammonia-oxidizing archaea abundance, (**e**) AOB abundance, and (**f**) their ratio. CK, control; U, urea; CSMP, CSMP fertilizer; U + CSMP, combined application of CSMP fertilizer and urea. Data are presented as means ± SE (n = 3). Different lowercase letters indicate significant differences among treatments (*p* < 0.05).

On day 3, relative to CK, CSMP had already elevated 16S rRNA gene copies, whereas U caused a decline in both acidic soils (Figure 3a). By day 30, compared with CK, gene abundance in CL had decreased by 5.30%, 3.02%, and 3.61%, while in WS it had increased by 1.12%, 1.53%, and 1.72%, respectively. The CSMP treatment significantly reduced ITS in both acidic soils on day 3 versus CK (Figure 3b). By day 30, the CSMP treatment increased fungal abundance in CL yet decreased it in WS relative to CK. In WS, the CSMP treatment also significantly lowered the ITS/16S ratio at both 3 and 30 days compared with CK (Figure 3c).

The AOB-*amoA* gene abundance responded more strongly to urea and CSMP applications compared with AOA-*amoA* (Figure 3d). Specifically, a significant increase of 0.44% in AOA-*amoA* gene abundance was observed in soil CL on day 3 after CSMP application compared with CK (*p* < 0.05). By day 30, urea significantly increased AOB-*amoA* gene abundance across all soil types relative to CK (*p* < 0.05), as depicted in Figure 3e. The CSMP treatment compared with CK induced significant increases in AOB-*amoA* gene abundance by 8% in CL and 40% in WS soils on day 30 (*p* < 0.05). Following the application of urea and/or CSMP, the AOA/AOB ratio significantly decreased in both soils on day 30 versus CK (*p* < 0.05), as illustrated in Figure 3f.

**Figure 4:**
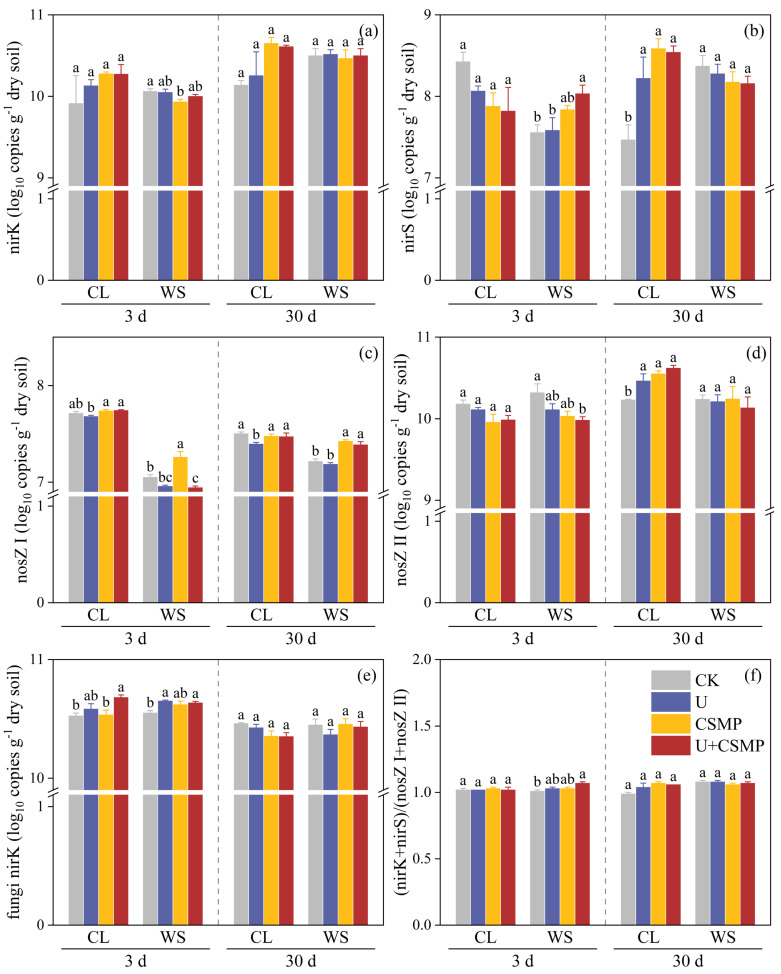
Effects of CSMP and urea application on microbial community structure in two acidic soils (CL and WS): (**a**) *nirK*, (**b**) *nirS*, (**c**) *nosZ* I, (**d**) *nosZ* II, (**e**) fungi *nirK*, and (**f**) (*nirK* + *nirS*)/(*nosZ* I + *nosZ* II). CK, control; U, urea; CSMP, CSMP fertilizer; U + CSMP, combined application of CSMP fertilizer and urea. Data are presented as means ± SE (n = 3). Different lowercase letters indicate significant differences among treatments (*p* < 0.05).

The initial incubation phase (day 3) revealed divergent responses of *nirK* and *nirS* genes in soil CL to all treatments. CSMP application, either alone or combined with urea, elevated *nirK* levels and reduced *nirS* levels compared with CK. By day 30, relative to CK, both *nirS* and *nirK* genes in soil CL showed a significant increase following treatments with CSMP and/or urea (*p* < 0.05), with CSMP alone causing the most substantial rise (Figure 4a,b). In contrast, compared with CK, soil WS showed a significant decrease in *nirK* gene abundance under the CSMP treatment at day 3 (*p* < 0.05), while the U + CSMP treatment significantly enhanced *nirS* gene levels (*p* < 0.05) (Figure 4b). At day 30, relative to CK, the application of urea and/or CSMP significantly increased the abundance of the *nosZ* II gene in soil CL (*p* < 0.05), and the *nosZ* I gene in soil WS also demonstrated a significant increase upon the CSMP treatment (*p* < 0.05) (Figure 4c,d). The fungal nitrite reductase gene *nirK* displayed significant variation only at day 3, with the U + CSMP treatment in soil CL and the U and U + CSMP treatments in soil WS both showing enhanced gene expression compared with CK (*p* < 0.05) (Figure 4e). In addition, the (*nirK* + *nirS*)/(*nosZ* I + *nosZ* II) in soil WS significantly enhanced at day 3 versus CK (*p* < 0.05) (Figure 4f).

### 3.4. Structural Equation Modeling

**Figure 5:**
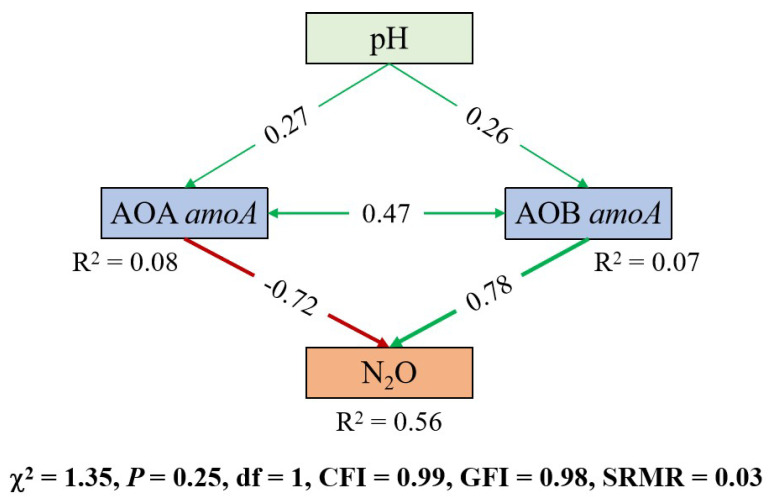
SEM illustrating the effects of amendments on N_2_O emissions. Green arrows indicate positive relationships, while red arrows indicate negative relationships (*p* < 0.05). The R^2^ values associated with response variables represent the proportion of variance explained by the model. The values on the arrows denote standardized path coefficients.

An SEM was constructed to clarify how soil N_2_O emissions are linked to pH shifts and to changes in the abundance of key functional genes in acidic soils (Figure 5). The model indicates that CSMP application significantly enhanced soil pH compared with initial levels, which subsequently stimulated the growth of both AOA (λ = 0.27) and AOB (λ = 0.26). However, their impacts on N_2_O emissions exhibited fundamentally distinct patterns: AOA abundance showed a negative correlation with N_2_O emissions (λ = −0.72), whereas AOB demonstrated a strong positive association (λ = 0.78). Notably, the model identified a synergistic interaction between AOA and AOB (λ = 0.47) that further increased this process.

## 4. Discussion

### 4.1. N_2_O Emission and N Transformations Following CSMP and Urea Application

The regulatory effects of alkaline amendments on N_2_O emissions from acidic soils have acquired increasing research attention [13,42,43]. Conventional theory proposes that lime-based amendments primarily reduce greenhouse gas emissions by elevating soil pH [9,43]. In the present study, CSMP application significantly increased soil pH in both acidic soils used (Figure S1). In these two moderately acidic soils, N_2_O emissions were dominated by bacterial nitrification. CSMP application, and especially the combined U + CSMP application, appreciably stimulated N_2_O emissions relative to the CK (Figure 1). Although earlier studies suggested that nitrification can be less intense in acidic soils [44], a recent meta-analysis showed that N_2_O emissions related to soil nitrification are higher in acidic soils compared with neutral and alkaline soils [45].

In the two acidic soils in the present study, CSMP markedly accelerated NH_4_^+^-N consumption versus CK (Figure 2a,b). This is because relatively high pH values stimulate the consumption of NH_4_^+^ in soils [23]. In the present study, accelerated NH_4_^+^-N consumption was accompanied by pronounced increases in net nitrification rates (up to 19.1-fold versus CK in WS soil, Figure 2f). These findings strongly suggest that CSMP influenced N_2_O emissions primarily through the stimulation of nitrification rather than by conventional denitrification pathways [27]. Nitrification generally acts as the primary process of N_2_O production in aerobic soils [46,47]. Therefore, an increase in the soil nitrification rate can directly promote higher N_2_O emissions in aerobic soil [48,49]. This explains why CSMP promoted both soil nitrification and N_2_O emission in the present study in soils in which N_2_O emissions were nitrification-driven.

The U + CSMP treatment, in particular, had pronounced effects on N_2_O production compared with other treatments (Figure 1). This synergistic effect appears to result from three key mechanisms: (1) Urea hydrolysis provided abundant NH_4_^+^ substrate for nitrification (Figure 2b). Plausible mechanisms for enhanced soil N_2_O emissions following urea addition may be enhanced NH_4_^+^-N/NO_3_^−^-N concentrations and stimulated N-cycling enzyme activities, thereby increasing N_2_O emissions [50]. (2) Transient pH spikes from urea hydrolysis complemented sustained alkalinity from CSMP addition [51]. (3) Substantial increases occurred in AOB-*amoA* gene abundance (40% versus CK in WS soil; Figure 3e) accompanied by decreased AOA/AOB ratios (Figure 3f), reflecting the predominance of bacterial ammonia oxidation [52,53].

### 4.2. CSMP Reduced the Acidic Soils’ N_2_O Emissions Dominated by Nitrification

Soil N_2_O production arises from the intricate interplay between nitrification and denitrification [54,55]. Increasing evidence indicates that nitrification-derived N_2_O emissions may actually exceed those from denitrification on a global scale, accounting for an average of 51.6% of total N_2_O emissions [56]. A study using ^15^N showed that, even under acidic conditions, nitrification can contribute more to N_2_O emission than denitrification [57]. pH is an important factor influencing the soil nitrification process [16]. This study observed an increase in the net nitrification rate of two acidic soils, compared with the CK, after alkaline amendment (Figure 2e,f). Following the addition of CSMP, soil H increased, which stimulated nitrification, which in turn, stimulated soil N_2_O emission.

The SEM identified AOB-*amoA* abundance as the key driver of N_2_O emissions in the acidic soils (Figure 5). While AOA typically mediate ammonia oxidation in unamended acidic soils and showed a stronger correlation with background N_2_O fluxes in this study [58], AOB exhibited greater responsiveness to pH elevation [53]. This aligns with reports that liming preferentially stimulates AOB-mediated N_2_O production due to their higher per-mole NH_3_ oxidation yield [34,42]. Consistent patterns emerge across ecosystems: Norwegian pH gradient experiments documented pH-dependent AOB enrichment (*p* < 0.001) concurrent with AOA decline (*p* = 0.02) [59], while meta-analyses indicate non-significantly greater liming effects on AOB versus AOA-*amoA* genes [60]. Biochar amendments in subtropical red soils similarly increased AOB abundance versus CK without affecting AOA, despite the numerical dominance of the latter [61]. These collective findings reveal a critical dichotomy: AOA govern nitrifier-derived N_2_O in native acidic soils, whereas pH-modifying amendments shift dominance to AOB. This mechanistic framework explains the contradictory biochar effects on N_2_O fluxes [62], where concurrent stimulation of AOB (increasing N_2_O) and *nosZ*-harboring denitrifiers (promoting N_2_O reduction) yields net effects dependent on their relative activation. However, this study acknowledges the inherent limitations of DNA-based approaches in quantifying microbial activity [63]. Future studies that pair transcriptional assays with isotopic tracing are required to rigorously test the proposed mechanism [64].

Such synergistic approaches offer the potential to maintain soil quality improvements while mitigating AOB-driven N_2_O emissions [65]. Despite limited existing research [66], this study proposes that coupling CSMP with approaches that mitigate nitrification could provide a dual-benefit strategy: sustaining soil-quality gains while suppressing GHG emissions [66]. This proposition is supported by previous studies in which lime was combined with DMPP (3,4-dimethylpyrazole phosphate), such as the 61.2% reduction in N_2_O emissions achieved relative to lime alone [67]. Future research could therefore consider the combined application of alkaline amendments with nitrification inhibitors to reduce soil N_2_O emissions and N losses while amending acidic soils.

## 5. Conclusions

This study demonstrated that CSMP exerted a strong yet context-dependent influence on soil N_2_O emissions in acidic soils. Contrary to the prevailing assumption that alkaline amendments universally mitigate N_2_O fluxes via enhanced bacterial N_2_O reductase abundance activity compared with CK, these results showed that CSMP application—particularly when co-applied with urea—substantially increased nitrification-driven N_2_O emissions by selectively stimulating AOB over AOA. This microbial pathway dominance compared with CK, as evidenced by elevated AOB-*amoA* gene abundance and suppressed AOA/AOB ratios, overrides the potential mitigating effects of increased *nosZ* II gene expression, thereby establishing pH-mediated AOB proliferation as the primary driver of emission intensification. Building on current field practices where nitrification inhibitors effectively reduce N_2_O emissions relative to fertilizers without inhibitors, the results suggest that combining CSMP with nitrification inhibitors could offer a promising dual-benefit solution for acidic soil management, simultaneously improving soil conditions while controlling N_2_O emissions. Future field studies should evaluate the feasibility of this integrated approach under realistic agricultural conditions.

## Data Availability

Data will be made available on request.

## Supplementary Materials

The following supporting information can be downloaded at: https://www.mdpi.com/article/10.3390/biology14091110/s1, Figure S1: (**a**) pH dynamics in soil CL, (**b**) pH dynamics in soil WS, all as influenced by CSMP and urea application. CK, control; U, urea; CSMP, CSMP fertilizer; U + CSMP, combined application of CSMP fertilizer and urea. Values are means ± SE (n = 3). Different lowercase letters indicate significant differences among treatments (*p* < 0.05); Table S1: physical and chemical properties of the test soil; Table S2: Elemental composition of silicon–calcium–potassium–magnesium fertilizer; Table S3: Primer sequences for PCR amplification. Refs. [68,69,70,71,72,73,74,75,76] are cited in Supplementary file.
