# Supplementary material for: Liming-Induced Nitrous Oxide Emissions from Acidic Soils Dominated by Stimulative Nitrification
Source: Biology (Basel). 2025 Aug 22;14(9):1110. doi: 10.3390/biology14091110 (PMC12467990; doi:10.3390/biology14091110)
Supplement: Supplementary file 1 [file biology-14-01110-s001.zip › biology-3800849-supplementary.pdf]

Table S1. Physical and chemical properties of the test soil.

Item	Clay (%)	Silt (%)	Sand (%)	pH	TN (g kg-1)	SOM (g kg-1)	C/N	Olsen-P (mg kg-1)	Available K (mg kg-1)
CL	13.5	57.4	29.1	5.81	2.05	18.6	9.06	40.7	46.7
WS	11.2	58.5	30.3	4.91	2.42	26.6	11.0	44.9	80.0

Table S2. Elemental composition of calcium-silicon-magnesium-potassium (CSMP) product.

Element	Content %	Compound	Content %
Ca	53.7	CaO	41.7
Al	7.85	SiO_2	27.8
Fe	5.66	K_2O	4.72
Si	18.4	MgO	1.87
K	6.50	Fe_2O_3	3.90
Mg	1.49	P_2O_5	1.46

Table S3. Primers sequences for PCR amplification.

Genes	Primer	Primer sequence (5'→3')	Prod-uct size	Reference
16S rRNA	338F<br>806R	ACTCCTACGGGAGGCAGCAG<br>GGACTACHVGGGTWTCTAAT	470bp	[68]
ITS	ITS1F<br>ITS2R	CTTGGTCATTTAGAGGAAGTAA<br>GCTGCGTTCTTCATCGATGC	268bp	[69]
AOA-*amoA*	amo19F<br>CrenamoA616r48x	ATGGTCTGGCTWAGACG<br>GCCATCCABCKRTANGTCCA	624bp	[70]<br>[71]
AOB-*amoA*	amoA-1F<br>amoA-2R	GGGGTTTCTACTGGTGGT<br>CCCCTCKGSAAAGCCTTCTTC	492bp	[72]
nirK	nirK876C<br>nirK1040	ATYGGCGGVCAYGGCGAA<br>GCCTCGATCAGRTTRTGG	164bp	[73]
nosZ I	nosZ2F<br>nosZ2R	CGCRACGGCAASAAGGTSMSSGT<br>CAKRTGCAKSGCRTGGCAGAA	267bp	[74]
nosZ II	nosZ-II-F<br>nosZ-II-R	CTIGGICCIYTKCAYAC<br>GCIGARCARAAITCBGTRC	698bp	[75]
Fungi *nirK*	338F<br>806R	ACTCCTACGGGAGGCAGCAG<br>GGACTACHVGGGTWTCTAAT	470bp	[76]

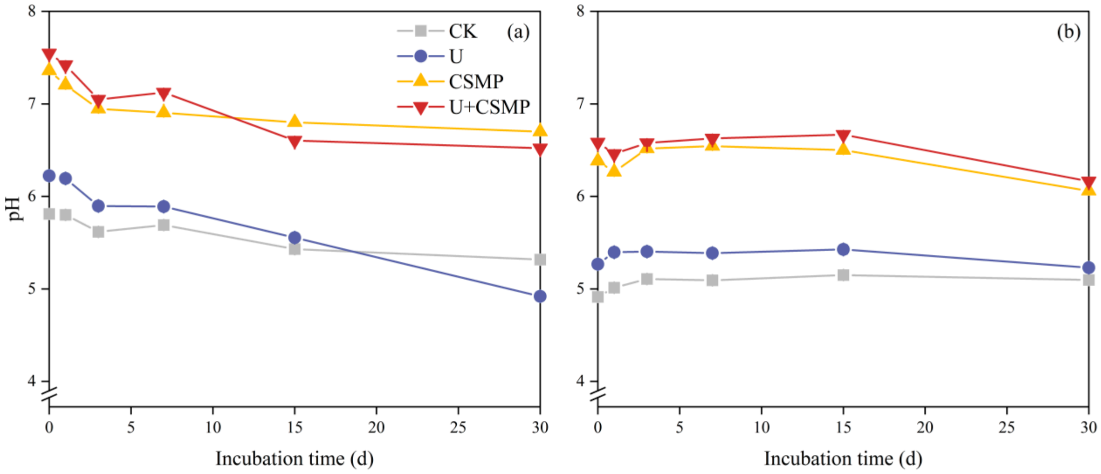

Figure S1. (a) pH dynamics in soil CL, (b) pH dynamics in soil WS, all as influenced by CSMP and urea application. CK, control; U, urea; CSMP, CSMP fertilizer; U+CSMP, combined application of CSMP fertilizer and urea. Values are means ± SE (n = 3). Different lowercases indicate significant differences among treatments ($P < 0.05$).

Reference

68. Mori, H.; Maruyama, F.; Kato, H.; Toyoda, A.; Dozono, A.; Ohtsubo, Y.; Nagata, Y.; Fujiyama, A.; Tsuda, M.; Kurokawa, K. Design and experimental application of a novel non-degenerate universal primer set that amplifies prokaryotic 16S rRNA genes with a low possibility to amplify eukaryotic rRNA genes. Dna Res 2014, 21, 217-227.
69. Yao, Q.; Liu, J.; Yu, Z.; Li, Y.; Jin, J.; Liu, X.; Wang, G. Three years of biochar amendment alters soil physiochemical properties and fungal community composition in a black soil of northeast China. Soil Biology and Biochemistry 2017, 110, 56-67.
70. Leininger, S.; Urich, T.; Schloter, M.; Schwark, L.; Qi, J.; Nicol, G.W.; Prosser, J.I.; Schuster, S.C.; Schleper, C. Archaea predominate among ammonia-oxidizing prokaryotes in soils. Nature 2006, 442, 806-809.
71. Schauss, K.; Focks, A.; Leininger, S.; Kotzerke, A.; Heuer, H.; Thiele Bruhn, S.; Sharma, S.; Wilke, B. M.; Matthies, M.; Smalla, K. Dynamics and functional relevance of ammonia - oxidizing archaea in two agricultural soils. Environ Microbiol 2009, 11, 446-456.
72. Rotthauwe, J.; Witzel, K.; Liesack, W. The ammonia monooxygenase structural gene *amoA* as a functional marker: molecular fine-scale analysis of natural ammonia-oxidizing populations. Appl Environ Microb 1997, 63, 4704-4712.
73. Harter, J.; Krause, H.-M.; Schuettler, S.; Ruser, R.; Fromme, M.; Scholten, T.; Kappler, A.; Behrens, S Linking N_2O emissions from biochar-amended soil to the structure and function of the N-cycling microbial community. The Isme Journal 2014, 8, 660-674.
74. Henry, S.; Bru, D.; Stres, B.; Hallet, S.; Philippot, L. Quantitative detection of the *nosZ* gene, encoding nitrous oxide reductase, and comparison of the abundances of 16S rRNA, *narG*, *nirK*, and *nosZ* genes in soils. Appl Environ Microb 2006, 72, 5181-5189.
75. Jones, C. M.; Graf, D. R. H.; Bru, D.; Philippot, L.; Hallin, S. The unaccounted yet abundant nitrous oxide-reducing microbial community: a potential nitrous oxide sink. The Isme Journal 2013, 7, 417-426.
76. Long, A.; Song, B. K.; Fridey, K.; Silva, A. Detection and diversity of copper containing nitrite reductase genes (*nirK*) in prokaryotic and fungal communities of agricultural soils. Fems Microbiol Ecol 2015, 91
